# Optimization of Betulinic Acid Extraction from *Tecomella undulata* Bark Using a Box-Behnken Design and Its Densitometric Validation

**DOI:** 10.3390/molecules21040393

**Published:** 2016-04-06

**Authors:** Nahida Siddiqui, Vidhu Aeri

**Affiliations:** Department of Pharmacognosy and Phytochemistry, Faculty of Pharmacy, Jamia Hamdard, New Delhi 110062, India; nahida62khan@gmail.com

**Keywords:** betulinic acid, Box-Behnken design, *Tecomella undulata*, high performance thin layer chromatography (HPTLC), Bignoniaceae

## Abstract

Betulinic acid (BA) is a pentacyclic triterpenoid acid obtained from the stem bark of *Tecomella undulata* Seem. (Bignoniaceae). Development of an efficient extraction method for the isolation of BA is important as it has a wide range of pharmacological activity. A Box-Behnken design (BBD) was used to investigate the effect of extraction variables such as temperature (30–60 °C), time (4–8 h) and solvent to drug ratio (300–500 mL/100 g) on the maximization of BA yield and its quantification using validated densitometric high performance thin layer chromatography coupled with ultraviolet detection (HPTLC-VIS). A quadratic polynomial model was found to best fit the model with *R*^2^ = 0.99. The optimized Soxhlet extraction yielded 2.449% *w/w* of BA at a temperature 53.86 °C, time 6.38 h and solvent to drug ratio 371 mL/100 g. BA in *Tecomella undulata* bark was detected at *Rf* value of 0.65 at 510 nm using the solvent system toluene–ethyl acetate–glacial acetic acid (8.5:1.5:0.02 *v/v/v*). The analytical method was validated and the linear regression analysis reflects good linear relationship (*R*^2^ = 0.9902). Lower %RSD and SEM suggested that the developed HPTLC-VIS method was precise, accurate and robust. Therefore, these economical techniques are very efficient and promising for the extraction and quantification of pharmaceutically important BA.

## 1. Introduction

**Figure 1:**
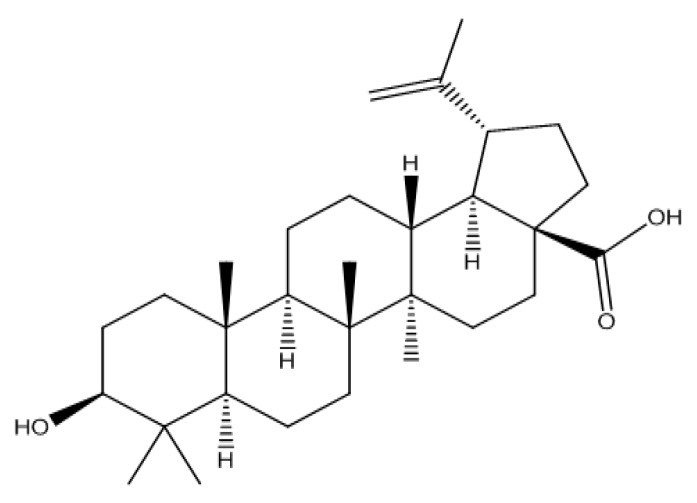
Chemical structure of BA (betulinic acid).

BA (3β-hydroxylup-20-(29)-en-28-oic acid, Figure 1) is a pentacyclic lupane type triterpenoid acid isolated naturally from the stem bark of *Tecomella undulata* belonging to the family Bignoniaceae [1]. Considerable amounts of BA (up to 2.5%) are available in the outer bark of a variety of tree species that are valuable for timber purposes [2]. As per the Ayurvedic Pharmacopoeia of India (2008 edition) *Tecomella undulata* is known as Rohitaka and used traditionally as a hepatoprotective agent in various ayurvedic formulations such as Rohitakarishta, Rohitakadya churna, Rohitaka ghrita and Rohitaka Lauha [3]. Recently Jain and coworkers proposed that the hepatoprotective potential of this plant is due to BA [4]. Many other authors have also suggested the hepatoprotective nature of this compound [5,6,7,8,9]. In addition to hepatoprotective activity, BA also shows a wide range of other pharmacological activities such as antihyperglycemic, antiobesity [10,11], anti-inflammatory [12], antineoplastic [13,14], antibacterial [15], anti-HIV and anthelmintic effects [2]. Due to its versatile pharmacological activity, it is an important constituent to isolate and analyze from natural sources using a simple, efficient and reliable technique. Conventional Soxhlet extraction (CSE) is used since a long time especially because of its low cost and easy applicability at laboratories as compared to the available novel techniques. Extraction of any kind of secondary metabolite by using wide range of solvents is the other advantage of Soxhlet extraction [16]. In present study, extraction of BA from the stem bark of *Tecomella undulata* was achieved by Soxhlet extraction which is a quite suitable mode for the extraction of non-polar metabolites.

Response surface methodology (RSM) is a more advantageous multivariate statistical technique than the traditional single parameter optimization in that it saves time [17]. It has been exploited for optimizing the extraction parameters of various phytocompounds like colchicine from *Gloriosa superba* [18], anthraquinones from *Rheum palmatum* [19], anthocyanins from *Ribes nigrum* [20] and anthocyanins, phenolic and total flavonoid content from *Vitis vinifera* [21]. More specifically, BBD is used in this regard. BBD is known to be more efficient than central composite and three level full factorial RSM designs because it allows estimation of the parameters of the quadratic model, building of sequential designs, detection of lack of fit of the model and use of blocks. Moreover, BBD has the desirable feature of needing the smallest number of experimental runs and it is useful in avoiding experiments performed under extreme conditions to provide unsatisfactory results. In this study, only 17 experiments are required for a three-factor, three-level study. It is also helpful in exploring of quadratic response surface and creating a second-order polynomial model. The experimental design consists of a group of experimental conditions lying at the centre of every edge and also the replicated centre point of the four-dimensional cube that defines the region of interest [22].

In the present study extraction parameters *viz*. temperature, time and solvent to drug ratio for the extraction of betulinic acid in hexane extract from the stem bark of *Tecomella undulata* were optimized employing BBD. Additionally, BA was quantified by a validated, simple and efficient HPTLC-VIS technique. This technique is extensively used because it is rapid, economical, sensitive, precise and has the capability to process a large number of samples in less time [23].

## 2. Results and Discussion

### 2.1. HPTLC-VIS Analysis of BA

The Soxhlet extraction technique was applied for the extraction of BA-containing hexane extract. The presence of BA in the hexane was confirmed extract by TLC by comparison with a standard. BA was quantified using a validated HPTLC-VIS technique for all experimental BBD runs.

**Figure 2:**
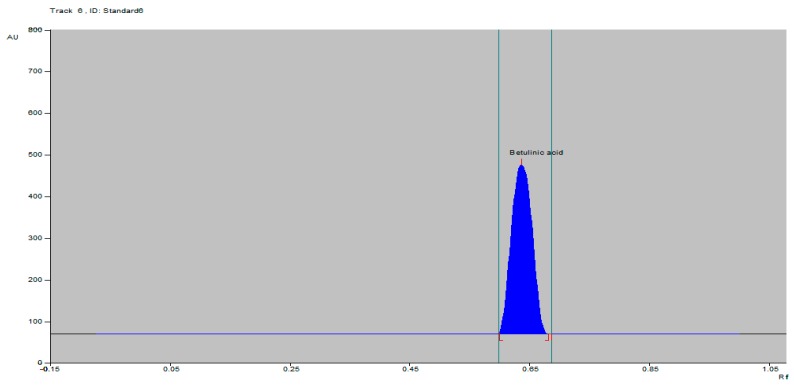
HPTLC chromatogram of BA standard.

**Figure 3:**
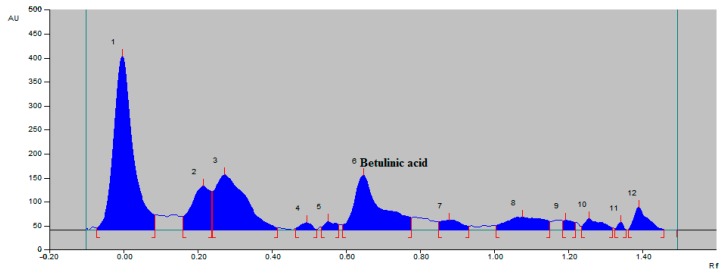
HPTLC chromatogram of BA in hexane extract of *Tecomella undulata* bark.

A solvent system consisting of toluene–ethyl acetate–glacial acetic acid (8.5:1.5:0.02 *v/v/v*) which provided a sharp and well-defined BA peak at an *Rf* value of 0.65 was selected (Figure 2). This solvent system for the isolation of BA has been reported herein for the first time. It was evident that the solvent system had a very good resolution for the separation of BA from the hexane extract of the *Tecomella undulata* bark (Figure 3).

**Figure 4:**
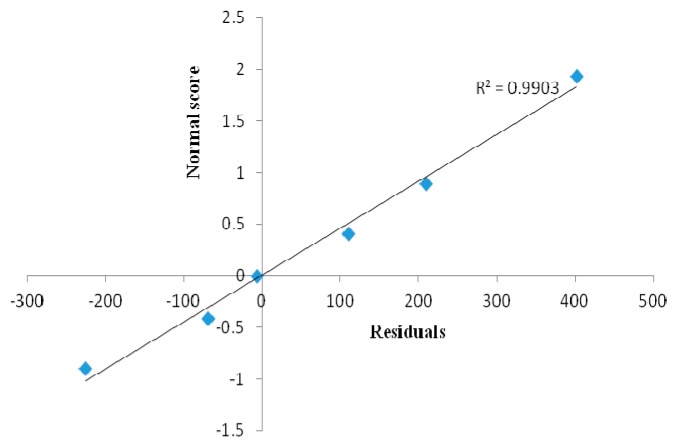
Normal distribution graph between residuals and normal score.

The linear regression data obtained for the calibration curves (*n* = 3) showed a good linear relationship over a wide range of concentrations (100–600 ng/spot) with respect to peak area (Table 1). The y-intercept of the linear equation was statistically insignificant, with a *p*-value of 0.20 (*p-*value > 0.05). Statistical analysis of the residuals was carried out at the level of confidence of 95% using Microsoft Office Excel 2010. The residuals have normal distribution (*R*^2^ = 0.9903) which is shown by a residuals *versus* normal score graph (Figure 4).

The skewness and kurtosis were found to be 0.28 and −0.19, respectively, which are within acceptable limits [24]. Validation parameters such as LOD and LOQ were found to be 6.37 and 19.32 ng, which indicates the sensitivity of method compared to other reported HPTLC methods for BA analysis [25]. The low value of %RSD and SEM suggests an excellent precision, accuracy and robustness of the developed method which are presented in Table 2, Table 3 and Table 4, respectively. The statistical analysis of the data indicates that the proposed method was reproducible, repeatable, selective, accurate and specific.

### 2.2. Model Fitting

The content of BA (% *w/w*) of each experimental BBD run was quantified using HPTLC-VIS and shown in Table 5. A quadratic model was found to be the best fit model and the comparative value for the proposed model is listed in Table 6. The value of “Adj R-Squared” was close to 1, indicating a high degree of correlation between the observed and predicted values. “Adeq Precision” measures the signal to noise ratio and it should be greater than 4 for model fitting. In this study, Adeq Precicion was found to be 41.12, indicating an adequate signal and that it can be used to navigate the design space. Table 7 showed the analysis of variance (ANOVA) for the extraction of BA from *Tecomella undulata*. The lack of fit F-value test for the model explains the deviation in the data around the fitted model. If the model does not fit the data well, the value of “Lack of fit” will be significant. In that case optimization of the fitted response surface is probably providing false results [23]. In this study, “Lack of Fit F-value” was found 0.93 which showed that the “Lack of Fit” is not significant relative to the pure error. Non-significant “Lack of fit” was good and it showed validity of RSM results. The Model F-value was 205.75 implies that the model was significant.

### 2.3. Effect of Extraction Parameters on BA Yield of Tecomella Undulata and RSM Analysis

Table 7 shows the ANOVA of the fitted quadratic polynomial model for BA yield. The linear variables (A, B, C), interaction variables (AB, BC, AC) and quadratic variables (A^2^, B^2^, C^2^) were found significant (*p* < 0.05). These data showed that the yield of BA was affected by all the variables as well as their combination and square of each variable. The *R*^2^ of the model was 0.99 and the coefficient of variation (%CV) was 1.11, showing a high degree of precision and a good deal of reliability of the experimental values [26]. The contributions of each independent variable are shown in Table 8.

Three-dimensional (3D) plots were constructed to visualize the relationship between independent variables and yield of BA according to the generated quadratic polynomial model equation of coded factors:

Yield of BA = +2.41 + 0.25·A + 0.096·B + 0.031·C − 0.048·AB + 0.031·AC − 0.039·BC − 0.27·A^2^ − 0.098·B^2^ − 0.15·C^2^ (1)

A positive value represents the a value in favor of the optimization, whereas a negative value indicates an inverse relationship between the independent variables and yield of BA. It is clear from the equation that variables such as temperature (A), time (B) and the solvent to drug ratio (C) had a positive effect on the yield of BA. It also showed that the relationship between response and variables was not always linear. When more than one variable is changed simultaneously, variables can produce different degrees of response. Interactions of A and B as well as B and C produced a negative impact on the response but A and C produced a favorable effect on the response. The result in the case of the square root of different variables produced different effects on the response which were not the same as shown by the individual variables in Table 8.

**Figure 5:**
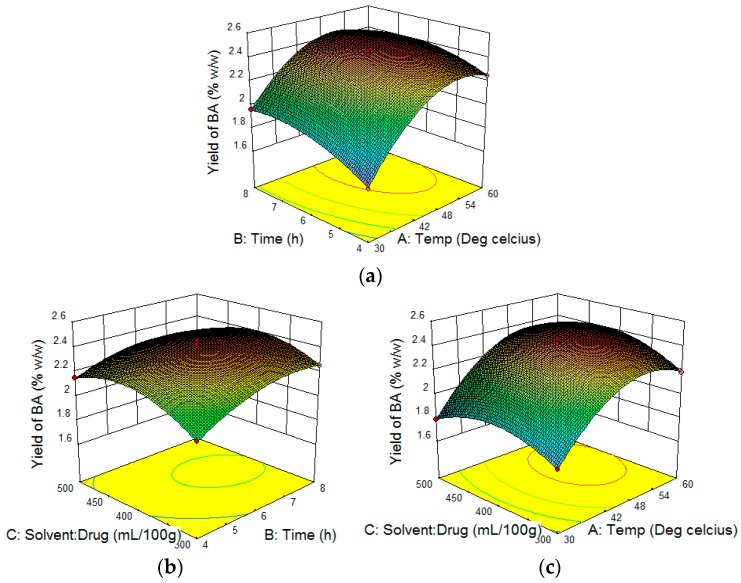
Response surface model 3D plots showing the effects of independent variable of Soxhlet extraction on BA yield: (**a**) temperature and time (**b**) time and solvent to drug ratio (**c**) temperature and solvent to drug ratio.

The square roots of A, B and C have negative effects on the response and among them A^2^ has more significant negative effect. This indicated that if temperature (A) were to be doubled then yield will be decreased strongly. The final combination ratio of the variables for the extraction was selected on the basis of yield of BA using three-dimensional response surface plots. Temperature (variable A) had a more significant effect on the yield of BA. As shown in Figure 5a,c, yield of BA was increased positively with extraction temperature up to 54.21 °C. However, the yield of BA decreased when the temperature was increased beyond 54.21 °C when solvent to drug ratio was fixed at 400 mL and time was set at 6 h. The maximum yield of BA was obtained at an optimum temperature of 54 °C, proving that a higher temperature is helpful in enhancing the compound yield as it increases the diffusion coefficient and solubility, although it may also cause compound degradation [27]. Figure 5b with the temperature set as 45 °C, showed an increased BA yield at longer time (variable B) and lower solvent to drug ratio (variable C). For BA yield, the optimum time was 6.38 h and the solvent to drug ratio was 369.25 mL/100 g.

### 2.4. RSM Validation

**Figure 6:**
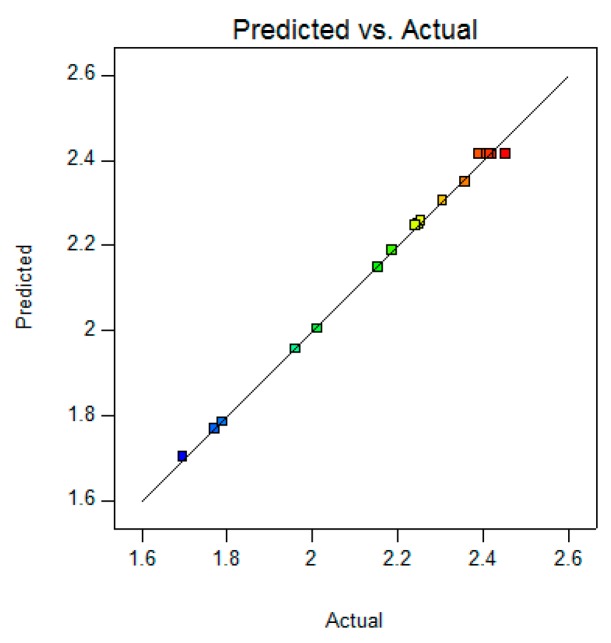
Linear correlation plot between actual and predicted values for the BA yield.

For the BA yield checkpoint, the yield evaluation result was found to be within limits. For validation of RSM results, the experimental values of the response were compared with the anticipated values and the percentage prediction error was found to be 1.67%. This is helpful in establishing the validity of the generated equation and to describe the domain of applicability of RSM model. The linear correlation plot drawn between the predicted and experimental value demonstrated a high *R*^2^ value (0.99), indicating excellent goodness of fit (*p* < 0.0001) (Figure 6). Thus the low magnitudes of error as well as the significant values of *R*^2^ in the present study prove the high prognostic ability of the RSM.

### 2.5. Optimization and Verification of the Model for Extraction Parameters

The optimum extraction process parameters were determined by maximizing the yield of BA. During the optimization stage, the desirability function of the Design-Expert™ (version 9.0.4) statistical software was applied to obtain the best compromise of response. The predicted optimal conditions for the extraction process were found at 54.21 °C temperature, 6.4 h time and 377.29 mL solvent and yield of BA was 2.456% *w/w*. The extraction process was again repeated by modifying the optimum extraction conditions *viz*. temperature 53.86 °C, time 6.38 h and the solvent to drug ratio was 371 mL. The BA yield was 2.449% ± 0.13% *w/w*. There was no significant difference (*p* > 0.05) between the predicted and experimental value, so this model may apply for the optimization of extraction process of BA from the stem bark of *Tecomella undulata*.

## 3. Experimental Section

### 3.1. Plant Material and Chemicals

Stem bark of *Tecomella undulata* was collected in April, 2014 from Lodhi Garden, New Delhi, India and was authenticated by a recognized taxonomist of National Institute of Science Communication and Information Resources, New Delhi, India (Ref no. NISCAIR/RHMD/Consult/2014/2472-51). A voucher specimen for the same has been deposited in the Department of Pharmacognosy and Phytochemistry, Faculty of Pharmacy, Jamia Hamdard, New Delhi, India for further reference. The stem barks were washed, dried and ground in a blender and stored in sealed plastic containers at room temperature. All the solvents used were of analytical grade and were purchased from Merck (Darmstadt, Germany). BA was purchased from Sigma Aldrich (St. Louis, MO, USA).

### 3.2. Soxhlet Extraction of BA

Hexane, chloroform, ethyl acetate and *n*-butanol extracts were prepared using Soxhlet extraction and 100 g of powdered stem bark of *Tecomella undulata* together with 300 mL of each organic solvent at 45 °C temperature for 6 h. Extracts were dried in a rotary evaporator under vacuum at 60 °C and TLC of each dried extracts were performed using a BA standard to determine its presence in the extracts.

### 3.3. Experimental Design

A preliminary study was performed to determine the presence of BA in the extracts of *Tecomella undulata*. BBD (Design Expert Software, Trial version 9.0.4.1, Stat-Ease Inc., Minneapolis, MN, USA) for RSM was applied with three factors, three levels and 17 runs for the optimization study of extraction variables viz. temperature, time and solvent to drug ratio to maximized the yield of BA. The proper ranges of the variables were determined according to single-factor experiments. All the samples were prepared and analyzed in triplicate. The independent and dependent variables are listed in Table 9.

An example of the polynomial equation generated by this experimental design is:

Response = X_0_ + X_1_ A + X_2_B +X_3_ C − X_4_ AB +X_5_ AC − X_6_ BC − X_7_ A^2^ − X_8_ B^2^ − X_9_ C^2^ (2)

where response was yield of BA (dependent variable) and A, B, C were the temperature, time and solvent to drug ratio (independent variables). X_0_ was the intercept and X_1,_ X_2_ and X_3_ are the linear regression coefficients. X_4_, X_5_ and X_6_ were the interaction coefficients and X_7_, X_8_, X_9_ were the squared coefficients.

### 3.4. HPTLC-VIS Analysis of BA

The 17 BBD runs of the hexane extracts (5 mg/mL) of *Tecomella undulata* bark were applied as spots (5 µL) on a silica gel aluminum plate 60F-254 (10 cm × 10 cm with 0.2 mm thickness, E Merck, Darmstadt, Germany) with a band width of 4 mm (Camag Linomat V, Muttenz, Switzerland). Prior to the application, the solutions were filtered through a 0.22 µm syringe filter using a Camag microlitre syringe. The space between two bands was 5.0 mm and the slit dimension was kept at 4 mm × 0.3 mm. 20 mm·s^−1^ scanning speed was used. The solvent system employed was toluene–ethyl acetate–glacial acetic acid (8.5:1.5:0.02 *v/v/v*). Linear ascending development was carried out in a twin trough glass chamber saturated with the mobile phase for 25 min at room temperature and the chromatogram was developed up to a height of 8.5 cm. The developed TLC plates were air dried for 30 min and then derivatized with anisaldehyde sulphuric acid reagent and dried in a hot air oven at 110 °C for 10 min. Densitometric scanning was performed using a Camag TLC scanner III in the absorbance mode at 510 nm. Concentration of BA in all the experimental runs were quantified by using regression equation obtained from the calibration curve of a BA standard.

### 3.5. Calibration Curve Preparation

A stock 1000 µg/mL solution of standard BA in HPLC grade methanol was prepared. Different concentrations of stock solution *viz*. 0.1, 0.2, 0.3, 0.4, 0.5, 0.6 µL were spotted in triplicate to obtain 100, 200, 300, 400, 500, 600 ng/spot concentrations of BA on silica gel plates. The data of peak area *versus* drug concentration was treated by linear least-squares regression.

### 3.6. Validation

The HPTLC method was validated according to the ICH guidelines [28].

#### 3.6.1. Accuracy

The recovery studies of BA were carried out by standard addition method which is spiking of different concentrations of BA in the hexane extract. The pre-analyzed sample of hexane extract was spiked with an extra 50%, 100% and 150% of the standard BA and the mixtures were reanalyzed by the proposed method. The experiments were conducted in triplicate. The %RSD and SEM of peak areas were calculated.

#### 3.6.2. Precision

The intra and inter-day variation of six replicates for the determination of BA were carried out at two different concentration levels of 200 and 400 ng/spot. The %RSD and SEM of peak areas were calculated.

#### 3.6.3. Robustness

By introducing small modifications in the solvent system, the effects on the result were examined. Solvent system having diverse composition of toluene–ethyl acetate–glacial acetic acid was tried at two different concentration levels of 200 and 400 ng/spot and %RSD of peak area was calculated.

#### 3.6.4. Limit of Detection (LOD) and Limit of Quantification (LOQ)

LOD and LOQ were calculated using Equations (3) and (4), respectively:

LOD = (3.3 × SD)/α(3)

LOQ = (10 × SD)/α(4)

where SD is the least standard deviation and α is the slope of the curve [29].

#### 3.6.5. Specificity

The specificity of the method was established by analyzing BA standard and its presence in hexane extract. The spot for BA in the extract was confirmed by comparing the *Rf* value, color and peak of the spot in samples with those of standard.

### 3.7. RSM Model and Validity Testing

Design-Expert™ software (version 9.0.4) was used to analyze the experimental results of the response surface design. A *p*-value less than 0.05 were considered to be significant. Independent variables of the extraction process such as temperature, time and solvent to drug ratio were simultaneously optimized by using RSM. Soxhlet extractions using the optimized conditions were performed in triplicate and the yield of BA was compared with the predicted values for the validation of the model.

## 4. Conclusions

The results showed that a validated HPTLC-VIS densitometric method and BBD can be very efficient and promising techniques for the identification and quantitative analysis of BA from *Tecomella undulata* bark. A BBD for RSM was used to estimate and optimize the experimental variables of extraction temperature (°C), time (h) and solvent to drug ratio (mL/100 g). All three variables, their interaction and the quadratic terms of each factor had a significant effect on the BA yield. A quadratic model for BA yield was derived with *R*^2^ = 0.99. The model prediction can be used optimize the yield of BA extractions from *Tecomella undulata* bark within the limits of the experimental variables. The modified optimal extraction conditions for BA in the bark extract were as follows: extraction temperature of 53.86 °C; extraction time of 6.38 h and solvent to drug ratio 371 mL/g. Under these conditions, the experimental result of BA yield was 2.456% ± 0.06% *w/w* which agreed closely with the predicted yield value. Linearity observed between the actual and predicted values of the response suggested the prognostic ability of the RSM design. 

The extraction of BA from the *Tecomella undulata* bark using Soxhlet extraction may be used as an alternative natural source of BA for pharmaceutical industries as it is in the development pipeline as an anticancer agent [2].

In this study a solvent system was developed for the first time for the HPTLC-VIS analysis of BA. The solvent system was found good for resolution of BA peaks. LOD and LOQ were found to be comparatively low which showed the great sensitivity of the developed method [25]. The statistical analysis of data indicates that the developed method is reproducible and specific.

## Figures and Tables

**Table 1:** Densitometric validation summary of betulinic acid.

Parameters	
Linearity range	100–600 ng
Correlation coefficient	0.9902
Regression equation (peak area)	Y= −174.788 + 3.090X
LOD (ng)	6.37
LOQ (ng)	19.32
*Rf* value	0.65
BA yield	2.4%

**Table 2:** Intra and Inter-day Method Precision (*n* = 6).

Amount of Betulinic Acid/Spot (ng)	Mean	SD	%RSD	SEM
Intra-day				
200	871.73	0.86	0.098	0.35
400	1317.12	1.06	0.080	0.43
Inter-day				
200	860.71	1.41	0.16	0.58
400	1328.073	1.74	0.13	0.71

**Table 3:** Accuracy Analysis (*n* = 3).

Amount of Betulinic Acid in Sample (mg)	% Age of Standard Betulinic Acid Added in a Sample	% Recovery of Betulinic Acid (Peak Area)	%RSD	SEM
0.06	0	98.69	0.5	0.29
0.06	50	97.44	1.05	0.59
0.06	100	97.58	1.27	0.72
0.06	150	99.26	1.03	0.59

**Table 4:** Robustness of Method (*n* = 3).

Betulinic Acid (ng/Spot)	Ratio of Solvent System	
	Toluene–Ethyl Acetate–Glacial Acetic Acid (8.2:1.8:0.02 *v/v/v*) %RSD via Area	Toluene–Ethyl acetate–Glacial Acetic Acid (8.5:1.5:0.02 *v/v/v*) %RSD via Area
200	0.16	0.57
400	0.075	0.49

**Table 5:** BBD Matrix for optimization of extraction of yield of BA.

Run	Temperature (A) (°C)	Time (B) (h)	Solvent to Drug Ratio (C) (mL/100 g)	Yield (Y) BA (% *w/w*)
01	60 (+1)	6 (0)	500 (+1)	2.305 ± 0.011
02	45 (0)	6 (0)	400 (0)	2.413 ± 0.003
03	30 (−1)	6 (0)	300 (−1)	1.771 ± 021
04	60 (+1)	6 (0)	300 (−1)	2.186 ± 0.008
05	45 (0)	8 (+1)	500 (+1)	2.241 ± 0.019
06	45 (0)	6 (0)	400 (0)	2.421 ± 0.045
07	30 (−1)	4 (−1)	400 (0)	1.696 ± 0.038
08	45 (0)	6 (0)	400 (0)	2.397 ± 0.098
09	60 (+1)	8 (+1)	400 (0)	2.358 ± 0.083
10	60 (+1)	4 (−1)	400 (0)	2.247 ± 0.051
11	45 (0)	6 (0)	400 (0)	2.451 ± 0.091
12	45 (0)	4 (−1)	500 (+1)	2.154 ± 0.004
13	30 (−1)	6 (0)	500 (+1)	1.789 ± 0.013
14	45 (0)	8 (+1)	300 (−1)	2.253 ± 0.021
15	45 (0)	6 (0)	400 (0)	2.388 ± 0.063
16	45 (0)	4 (−1)	300 (−1)	2.011 ± 0.051
17	30 (−1)	8 (+1)	400 (0)	1.961 ± 0.078

**Table 6:** Result of regression analysis for model and response regression equation for the final proposed model.

	Model F Value	*R*^2^	Adjusted *R*^2^	Predicted *R*^2^	SD	CV%
	Linear	0.5287	0.4199	0.2828	0.20	-
Yield of BA	2F1	0.5464	0.2742	−0.2106	0.22	-
	Cubic	0.9978	0.9911	-	0.024	-
	Quadratic	0.9962	0.9914	0.9717	0.024	1.11

**Table 7:** ANOVA for the fitted quadratic polynomial model of BA extraction.

Source	Sum of Square	Degree of Freedom	Mean Square	F-Value	Prob > F
Model	1.07	9	0.12	205.75	<0.0001 Significant
Residual	4.05	7	5.78		
Lack of fit	1.67	3	5.56	0.93	0.5030 Non significant
Pure error	2.38	4	5.96		

**Table 8:** Estimated regression model of the relationship between yield of BA and independent variables (A, B, C).

Variable	Degree of Freedom	Sum of Square	F-Value	*p* Value
AB	1	9.22	15.92	0.0053
AC	1	3.84	6.64	0.0366
BC	1	6.01	10.38	0.0146
A^2^	1	0.31	528.89	<0.0001
B^2^	1	0.04	70.41	<0.0001
C^2^	1	0.09	165.61	<0.0001

**Table 9:** Extraction variables selected for BBD optimization.

Independent Variable	Ranges of Independent Variable		Dependent Variable	Goal
	Low (−1)	High (+1)		
Temperature (°C)	30	60	Yield of BA (% *w/w*)	Maximized
Time (h)	4	6		
Solvent to drug ratio (mL/100 g)	300	500		
